# Intracystic Hemorrhage From a Splenic Artery Within a Pancreatic Pseudocyst: A Case Report

**DOI:** 10.7759/cureus.68897

**Published:** 2024-09-07

**Authors:** Bruna Oliveira Trindade, Fábio Herrmann, Matheus Biolchi, Rone Clayton, Mohamad Hamaoui, Mayara Christ Machry, Rodrigo Mariano, Angélica Lucchese

**Affiliations:** 1 Medical School, Federal University of Health Sciences of Porto Alegre, Porto Alegre, BRA; 2 Gastrointestinal Surgery, Santa Casa de Porto Alegre Hospital, Porto Alegre, BRA; 3 Medical School, Metropolitan Union of Education and Culture (UNIME - Lauro de Freitas/Bahia), Lauro de Freitas, BRA

**Keywords:** angiographic embolization, case report, intracystic hemorrhage, pseudocyst, surgery

## Abstract

A pancreatic pseudocyst, typically resulting from acute pancreatitis, is a cystic lesion that lacks a true epithelial layer and can lead to various complications, including hemorrhage, which is most often associated with the splenic artery. Hemorrhage within a pseudocyst is a rare but severe complication, manifesting as intracystic, peritoneal, or gastrointestinal tract bleeding. We present a unique case of a 50-year-old male farmer with a history of acute pancreatitis who developed an intracystic hemorrhage due to ischemia in the splenic artery traversing a pancreatic pseudocyst. The patient was successfully treated with angiographic embolization after presenting with symptoms of gastrointestinal bleeding, hypotension, and abdominal pain. Initial management included conservative monitoring, but upon further complications, intervention became necessary. The patient's postoperative course was uneventful, and follow-up imaging confirmed the resolution of the hemorrhage and stabilization of the pseudocyst. This case underscores the importance of recognizing and promptly treating hemorrhagic pancreatic pseudocysts, particularly those involving visceral vessels. It also highlights the role of angiographic embolization as an effective treatment option. Given the rarity of such cases, our report aims to contribute to the growing body of literature and provide guidance for the management of similar cases in the future. Continued documentation and study of these cases are essential to developing standardized treatment protocols and improving patient outcomes.

## Introduction

A pancreatic pseudocyst is the formation of a cystic lesion from reactive granulation tissue without a true epithelial layer which accumulates pancreatic fluid [1,2]. Regarding the etiology of pancreatic pseudocysts, acute pancreatitis is the main cause, followed by abdominal trauma, and abdominal surgery, among others [3]. Generally, pancreatic pseudocysts have a low incidence and regress spontaneously, although patients with symptoms associated with pseudocysts should undergo drainage [2].

Hemorrhage is one of the most threatening complications of pancreatic pseudocyst and predominantly involves the splenic artery [4]. As a consequence, it can present as intracystic, peritoneal, pancreatic duct, retroperitoneum, or digestive tract bleeding [5]. Although hemorrhage generally results from the erosion of an artery in the wall of the cyst, in some cases, the vessel might actually traverse the cyst cavity [6].

The current study presents the case of a splenic artery present within a pancreatic pseudocyst of a 50-year-old man, which was successfully treated by embolization.

## Case presentation

A 50-year-old male farmer with a history of acute pancreatitis, who was being followed in outpatient care for a big pancreatic pseudocyst, came to the hospital presenting nausea, vomiting, difficulty eating, and abdominal distension. On physical examination, a globular abdomen and a mass in the upper abdomen were noted.

**Figure 1:**
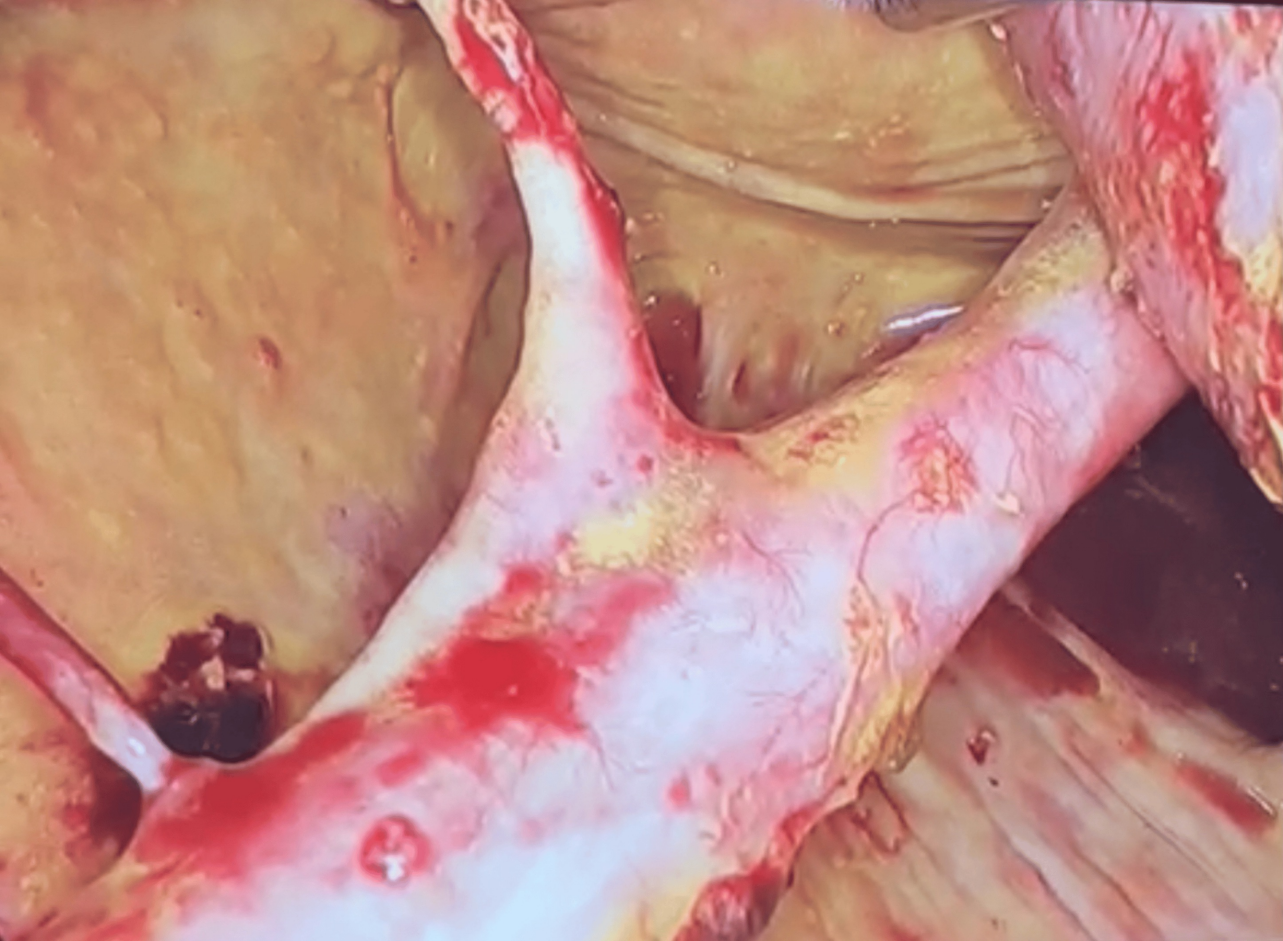
Splenic artery within the pancreatic pseudocyst

The patient underwent laparoscopic gastric-cyst anastomosis, with the anterior and posterior stomach walls being opened to access the pseudocyst. The posterior opening was about 4 cm. An anastomosis was performed with 3-0 polydioxanone suture (PDS), aspirating 10 liters of citrine liquid. The procedure was without complications. Additionally, the splenic artery was observed inside the cyst, and it was decided not to address the artery (Figure 1).

On the eighth day postoperatively, the patient presented with vomiting and abdominal distension. A CT scan showed an area of discontinuity in the posterior wall of the gastric body communicating with a heterogeneous gas-liquid collection occupying the body and tail of the pancreas, measuring 19.4 x 6.7 x 18 cm, with a volume of 1216 ml, and moderate to severe pneumoperitoneum in the upper abdomen, possibly due to the accumulation of gastric content inside the pseudocyst.

Due to the patient's stability, the team decided on conservative management with repeated CT scans. On the 22nd day after surgery, the collection size decreased significantly to 16.6 x 6.6 cm. The patient was discharged from the hospital after 23 days postoperatively due to clinical improvement.

Eight days post discharge, the patient returned to the hospital due to hematemesis and melena that started the same day, with an episode of syncope at night and associated abdominal pain. In the emergency room, the patient presented hypotension (blood pressure 84/44 mmHg) and a painful abdomen on palpation without signs of peritoneal irritation. On admission, a hemoglobin level of 4.4 was noted, and a rectal exam revealed feces with streaks of blood. Initial management of hemorrhagic shock was performed, and an upper gastrointestinal endoscopy showed no signs of bleeding. The CT scan did not show active bleeding, only a collection in the pseudocyst topography in the left hypochondrium, inferior to the gastric body, measuring 14.6 x 5.1 x 15.7 cm, with an approximate volume of 607 cm³, similar to the previous exam, with no free fluid in the cavity. The emergence of hypodense areas at the periphery of the spleen of ischemic nature was observed. The splenic artery showed filiform and irregular flow along its path through the area of the pancreatic pseudocyst. Adequate flow was not visualized in the splenic vein, which also traverses the pseudocyst.

**Figure 2:**
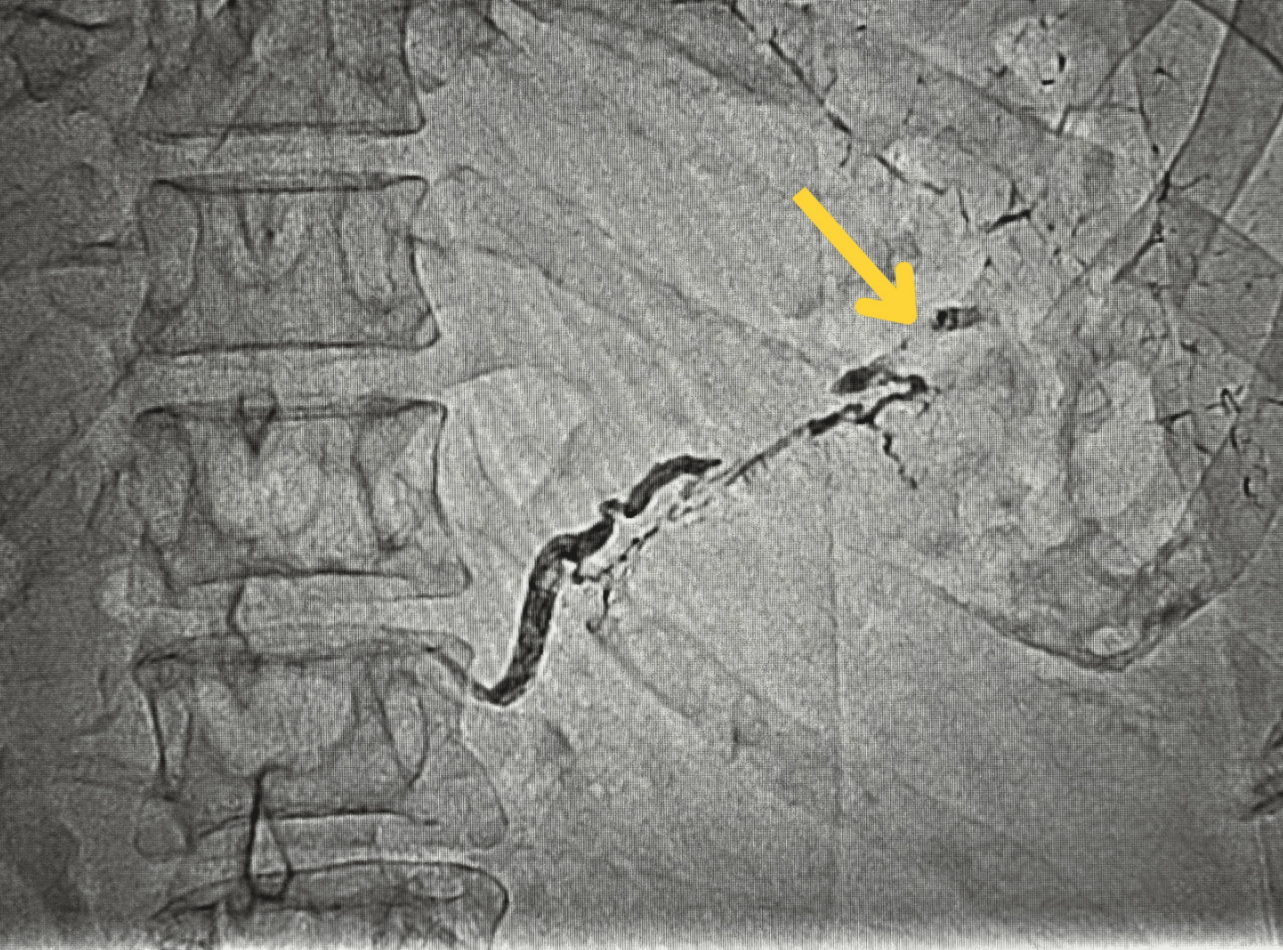
Arteriography showing splenic artery within the pancreatic pseudocyst Arrow shows the embolization site

On the same day, arteriography and angiographies of the superior mesenteric, inferior mesenteric, and celiac trunk arteries were performed, identifying an irregular and tapered splenic artery without active bleeding, which was embolized (Figure 2). The patient recovered with no complications in the postoperative period and was discharged with outpatient follow-up after significant improvement in the general condition, and a control CT scan showed a blocked collection.

## Discussion

Bleeding pseudocyst as a consequence of pancreatitis is a severe complication and can lead to massive gastrointestinal bleeding [7]. One mechanism of hemorrhage involves the inflammatory process, where the pseudocyst might cause compression or thrombosis in the portal and splenic veins, leading to localized portal hypertension [7,8]. The bleeding can be categorized into three types: intracystic hemorrhage, bleeding into the gastrointestinal tract, and expansion into the abdominal cavity [7,8]. 

The presented case exemplifies intracystic hemorrhage due to an ischemic process in the intracystic splenic artery. The development of bleeding complications undoubtedly necessitates radiological or surgical intervention [7]. In this case, angiographic embolization was chosen due to the patient's stability. Literature indicates that angiography allows embolization of the bleeding vessel, offering the advantage of being a single treatment option for inoperable patients and serving as a stabilizing measure before definitive surgery if needed [9,10].

Hemorrhage from this type of pseudocyst is rare and potentially fatal [9-11]. This is an acute complication that, according to the literature, most often results from a splenic artery pseudoaneurysm [10]. However, the presence of visceral vessels within the pseudocyst makes it even more critical to report such cases. A literature review on PubMed revealed only one case of visceral vessels within a pancreatic pseudocyst, where the vessel ran through the cavity without connection to the pseudocyst wall, but it was a pseudoaneurysm [6]. In terms of management, Chlorogiannis et al. reported a case of hemorrhagic giant superior mesenteric artery pseudoaneurysms located inside a pancreatic pseudocyst, which was successfully treated with endovascular repair [12]. Their patient experienced no complications and was discharged with complete resolution of bleeding. Therefore, less invasive treatments may be a viable alternative for managing bleeding in pancreatic pseudocysts.

We present this therapeutic approach to enhance the existing literature and support the scientific community in managing similar cases in the future. Knowing the variability of the artery course increases the awareness of radiologists and surgeons in diagnosis and surgical intervention which is of clinical significance to increase the quality of health services and decrease the iatrogenic faults [13]. Due to the current lack of long-term evidence on this specific technique, we emphasize that surgeons should continue to make decisions on a case-by-case basis. As more reports and comprehensive studies are conducted, we hope to establish stronger evidence for the routine application of this procedure.

## Conclusions

Hemorrhagic pancreatic pseudocysts, though rare, pose significant clinical challenges due to their potential severity. Our case emphasizes the critical need for individualized treatment plans, highlighting the efficacy of angiographic embolization in preventing further complications. It is essential to continue reporting similar cases and conducting comprehensive studies to help establish stronger evidence for standardized protocols, ultimately improving patient outcomes. We hope this report contributes valuable insights to the medical community and encourages further research into the management of hemorrhagic pancreatic pseudocysts
